# Mapping-by-Sequencing via eBSRmap (Easy Bulk Segregate RNA Mapping) in a B73 EMS Mutant Population

**DOI:** 10.3390/genes16111337

**Published:** 2025-11-06

**Authors:** Yang Cui, Jing Yang, Haiming Zhao, Hainan Zhao, Xiangbo Zhang

**Affiliations:** 1 Frontiers Science Center for Molecular Design Breeding (MOE), State Key Laboratory of Maize Bio-Breeding, Key Laboratory of Genome Editing Research and Application, Ministry of Agriculture and Rural Affairs, National Maize Improvement Center, College of Agronomy and Biotechnology, China Agricultural University, Beijing 100193, China; september516@163.com; 2 Sichuan Academy of Agricultural Sciences Rice and Sorghum Research Institute, Deyang 618000, China; 3 Center for Agricultural Genetic Resources Research, Shanxi Agricultural University, Taiyuan 030000, China; yangjingsx2025@163.com; 4 College of Agronomy and Biotechnology, China Agricultural University, Beijing 100193, China; haiming223@163.com; 5 Crop Research Institute, Guangdong Academy of Agricultural Sciences, Guangzhou 510640, China

**Keywords:** maize, Kernel traits, EMS mutant, RNA-seq, SNP, candidate genes, gene mapping, eBSRmap

## Abstract

Background: Maize, crucial for food, feed, and industry, is a model for genetic and breeding research. Kernel traits directly affect maize yield. This study developed the eBSRmap method to simplify gene cloning related to maize kernel traits. Methods: The eBSRmap method constructs a maize EMS mutant population, then conducts RNA-seq on pooled mutant and wild-type samples to identify SNP markers and map candidate genes for kernel trait mutations. Results: Applied to a maize EMS mutant population, eBSRmap identified candidate genes for twenty kernel trait mutants, successfully mapped twelve, and eight were confirmed by co-segregation analysis (success rate: 40%). The identified genes showed mutations like missense and stop-gained, related to phenotypes such as small, shrunk, and defective kernels. Conclusions: eBSRmap offers a fast and affordable way to map genes and identify candidate genes in a large-scale mutant population, aiding the understanding of gene functions in maize. The identified candidate genes can be further validated by functional analysis, which is significant for maize breeding and genetic improvement.

## 1. Introduction

Maize is an indispensable food, feed, and industrial crop as well as a classical model for genetics and breeding. Gene cloning serves as the pivotal bridge between basic research and applied breeding on maize. By resolving the genetic determinants of kernel size, weight, quality, and stress tolerance into individual genes and alleles, it transforms once-intractable traits into defined molecular pathways—illuminating how carbon is partitioned, how endosperm cells divide, and how starch and storage proteins are synthesized. These cloned genes can be translated into functional markers for marker-assisted and genomic selection, eliminating years of phenotypic cycling and accelerating cultivar development.

Map-based (positional) cloning—identifying causal DNA from mutant phenotypes via genetic recombination—remains a gold standard for gene discovery. Its first success in a human was isolating a gene via a ~500 kb chromosome walk [1]; arabidopsis followed via RFLP/YAC end-walking [2,3]. Instead of scarce and labor-heavy RFLP markers, SSR markers later emerged as a breakthrough, offering higher polymorphism, PCR-based efficiency, and superiority for maize mapping [4], enabling rapid mutant cloning [5,6,7].

Maize cloning was long hindered by its large genome (relying on transposon tagging/homology methods) until the BAC-based physical map and B73 reference genome [8]. Post 2009, standard map-based cloning narrowed targets to <200 kb via high-density markers (SSR, CAPS, InDel) before candidate gene mining [9,10,11,12,13].

“Mapping-by-sequencing”: SNP arrays [14,15], GBS [16,17], and bulked-segregant sequencing (MutMap, QTL-seq, 20–30× depth) deliver sub-centimorgan intervals in one generation [18,19,20]. Marker technology evolution (RFLP→SSR→SNP/GBS→bulked sequencing) has resolved bottlenecks, accelerating novel gene isolation.

We wondered whether gene cloning could be further simplified, eliminating the need for both a segregating population and whole-genome sequencing, capturing the mutant gene directly. Here, we present a method called Easy Bulked sergeant RNA-seq Mapping (eBSRmap). In maize, causal genes cloned underlying kernel traits consistently, showing a seed-dominant expression profile. These genes exhibit their highest transcript levels in developing kernels, peaking near 12 days after pollination (DAP), For example, Dek407 shows peak expression in the 12 DAP endosperm, whereas ZmABI19 peaks in the 12 DAP embryo [21,22]. Therefore, in principle, the causal mutant of an EMS-induced kernel variant should be detectable at the transcript level in the developing kernel. Based on this fact, eBSRmap can be used to further simplify gene cloning in large EMS mutant population. We screened 20 EMS-induced maize kernel mutants and identified candidate genes, eight out of twelve candidate genes were confirmed by co-segregation analysis. While it has certain limitations—such as a relatively low mapping success rate—eBSRmap has demonstrated its capability to handle large-scale mutation populations and still holds room for optimization.

## 2. Materials and Methods

### 2.1. Plant Materials

Mature pollens of inbred line B73 were mutagenized by immersing panicles in 0.015% of ethyl methane sulfonate (EMS) solution (*v*/*v*), with a ratio of pollen/solution = 1:10 for 10~15 min. Then the treated pollens were provided to the B73 ear. The resulting M1 plants were self-pollinated and the M2 kernels were obtained and identified by phenotype. M2 plants were further self-pollinated to obtain M3 progeny for the bulked pool. The plants were grown in the experimental field at the Shangzhuang Experimental Base of China Agricultural University under natural conditions (Beijing, China).

### 2.2. Classification of Kernel Phenotype

Referring to M.G. Neuffer’s maize kernel mutant classification method and other related literature about gene mapping based on maize kernel mutant, the mutant phenotypes are divided into five classes [23,24,25]: smk (small kernel), sh (shrunken), mn (miniature seed slightly loose pericarp), dek (defective kernel), and emp (empty pericarp).

### 2.3. cDNA Library Preparation and Data Analysis

Bulked kernels without pericarps were grinded into fine powder in liquid nitrogen. 50~100 μL of powder were placed in 1.5 mL of RNase-free centrifuge tubes and extracted with 1 mL of TRIzol™ Reagent (Thermo Fisher Scientific, Eugene, OR, USA; catalog no. 15596018).

cDNA libraries were prepared with VAHTS mRNA-seq v2 Library Prep Kit for Illumina^®^ (cat. no. NR601; Vazyme Biotech Co., Ltd., Nanjing, Jiangsu, China) and sequenced by the Illumina X-ten platform. Then, the yielded raw reads were aligned to B73 reference (V4) using hisat2 with the default parameters. The alignment files were converted to BAM format and sorted with the help of Samtools. SNP calling was performed by analyzing the unique reads with Samtools and bcftools. High-confidence SNPs were selected by using the following criteria: (1) an SNP site covered at least five reads, (2) SNP was detected in both mutant and its corresponding wild type, (3) SNPs that presented in more than 10% of lines were discarded. Functional annotation of SNPs was performed using SnpEff (Switch Laboratory, VIB—University of Leuven, Leuven, Belgium).

### 2.4. Statistical Simulation of D-Value Threshold

To determine the appropriate statistical threshold for D-value in BSA analysis, we performed Monte Carlo simulations based on the genetic structure of our EMS mutant population. The simulation modeled a single causal SNP with expected genotype frequencies of 100% homozygous mutant in the mutant pool and a 2:1 ratio of heterozygous to homozygous wild-type in the wild-type pool (theoretical D-value = 0.333). We simulated 100,000 replicates of bulk sequencing with pool sizes of 20 individuals each, incorporating both sampling error (binomial distribution) and technical variation (Poisson-distributed sequencing depth with λ = 30, plus normally distributed noise with SD = 0.05). The 95th percentile of the resulting D-value distribution was used as the statistical threshold for candidate gene detection.

### 2.5. Analysis of SNP Linkage

After filtering the high confidence SNP, we proceeded to access the degree of linkage by calculating the ratio of mutant base (hereafter named SNP index) at each SNP location for wild-type and the corresponding mutants, respectively. Moreover, the difference between the two SNP indexes (hereafter named D-value) were used as an important indicator to evaluate the linkage state. While the D-value is 0.4, the gene covered with such SNP would be recognized as the preliminary candidate.

### 2.6. Validation of Co-Segregation

To confirm the conferred genes responsible for the aberrant kernels, we designed corresponding primers (Table 1) to distinguish the genotype based on the candidate gene information. The primers were located at 105,751 bp upstream or downstream from the hotspot SNP. More than ten M3 mutant kernels were peeled of their pericarp and their DNA were extracted individually. Then we tested their genotype using the primers we have designed. If the genotype matched the phenotype well, these candidate genes will be recognized as the causative genes.

### 2.7. Allelic Test

Luckily, we found that two different mutants have the same candidate gene in our EMS library and both of them have similar kernel appearance. Therefore, to confirm the reliability of our methods, allelic test was performed in the following. As we cannot search any imprinting information about these genes, we proceeded to observe the progeny from the one randomly selected mutant as the mother, and the another as the father.

### 2.8. Validation by CRISPR/Cas9 Technologies

We selected several genes that passed the verification of co-separation and could not find other alleles in our EMS library to perform knock-down by CRISPR/Cas9 technologies in the LH244 background. Two proper 20 bp single guide RNA for one gene was selected, synthesized, and cloned into the CRISPR/Cas9 expression vector (pXUE411C-BG) according to published pipeline. EHA105 agrobacterium cells containing the target vector were infiltrated into 12 DAP embryos (1.5–1.8 mm). Then the selection media and regeneration media will be used to induce embryos to grow plants successfully. Herbicide resistance and DNA sequencing will be performed in the seedling stage to filter the positive transgene plants.

### 2.9. Accession Numbers

MN6 (Zm00001d037926); MN7 (Zm00001d003621); DEK51 (Zm00001d013056); ZmPPR278 (Zm00001d015156); HSP90.6 (Zm0000d041719); ZmEMP4 (Zm00001d033869).

### 2.10. Primers in This Study

The primers applied to amplify the mutation sites and flank sequences in this study are detailed in Table 1.

**Table 1:** Primers used for amplifying the mutation sites and flank sequences.

Mutant ID	Primer ID	Primer Sequence	Amplification Interval
#105	CY16_105CGF	5′-TGTGGAATGAGATGGAAAGTGC-3′	chr1: 276804254–276804810
	CY16_105CGR	5′-CCGCAGATGCCTCTTTTTC-3′	
#119	CY16_119CGF	5′-TGATTGCCCTTTACTCTATTGGGTA-3′	chr3: 134956480–134957182
	CY16_119CGR	5′-GTAACGCAACTTATCAAGAGCATCA-3′	
#180	CY16_180CGF	5′-CCATTGGACTCCCCTTTTGCT-3′	chr8: 14483769–14484442
	CY16_180CGR	5′-AACAGGGCAAGTATTTCATGG-3′	
#205	CY16_205CGF	5′-TATTGGGAGGAAAAATGATGCG-3′	chr5: 41112547–41112992
	CY16_205CGR	5′-GACAATACTTGAGTGCCTACTGAAT-3′	
#206	CY16_206CGF	5′-TGATAGGAGGACAGAGGGAAAG-3′	chr2: 50703053–50703671
	CY16_206CGR	5′-CAAAGTGAAGTCAGAACAGCA-3′	
#225	CY16_225CGF	5′-CGGCTCCACGGTAAAAATCAT-3′	chr5: 77316940–77317587
	CY16_225CGR	5′-CCCTTCTCTGTTCCCCATTCT-3′	
#226	CY16_226CGF	5′-TGATAGGAGGACAGAGGGAAAG-3′	chr2: 50703053–50703410
	CY16_226CGR	5′-TGAACTCTCACTGACTGCCGTAG-3′	
#254	CY16_254CGF	5′-TTCACAGCAAGCCCAAGACCG-3′	chr7: 2078837–2079339
	CY16_254CGR	5′-AAAAAAGGGCAAGGGTCAGAT-3′	
#2	CY16_2CGF	5′-CATAATCCGAATCAAGACAACCC-3′	chr5: 74947055–74947634
	CY16_2CGR	5′-ACTGTATTCTCCTTGGGTCATCTCC-3′	
#32	CY16_32CGF	5′-TTGAGTTACTTCAGTTGATGCC-3′	chr6: 142178366–142178996
	CY16_32CGR	5′-GGACTATGCTCTTCATTGTGTTTG-3′	
#43	CY16_43CGF	5′-ACTGCTCTGTTTGATTTTAGTGCTG-3′	chr5: 3927508–3928212
	CY16_43CGR	5′-CCAATGACATATCCGAGAGTTT-3′	
#82	CY16_82CGF	5′-TTCAACAACTTCTTAGCCGCCTTAC-3′	chr5: 77316835–77317586
	CY16_82CGR	5′-CCCTTCTCTGTTCCCCATTCT-3′	

## 3. Results

### 3.1. Construction of B73 EMS Mutant Population and Bulked Sergeant Library

**Figure 1:**
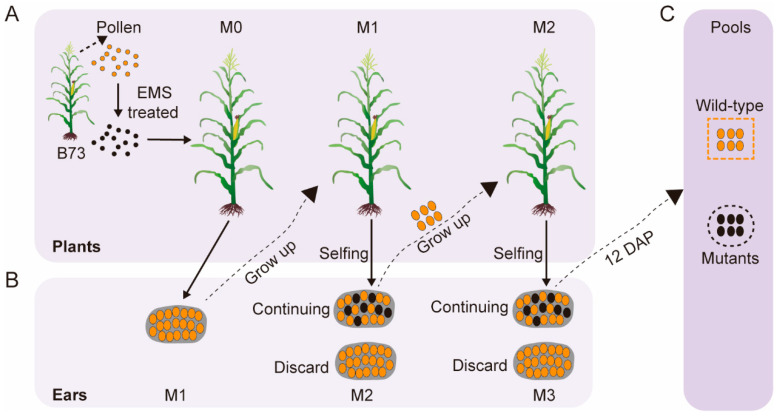
Construction of B73 EMS mutant population. (**A**,**B**): The pipeline for EMS-induced mutagenesis. The pollen from B73 is collected and treated with 0.015% EMS solution, which is provided to the unpollinated B73 ears. The resultant ears are named as M1. If kernels from the self-pollinated M1 are separate, such ears will be kept for further selfing. And if not, it will be discarded. Then the normal-like M2 will repeat the process that their parents had experienced in the previous generation. Finally, 20 normal kernels and its abnormal siblings on the M3 ear will form a wild-type or mutant pool, respectively. (**C**): The ellipses colored with orange or black represent the kernels that are normal or defective, respectively. Color coding: yellow indicates wild-type pollen or seeds, while black indicates mutant pollen or seeds.

Elite maize inbred line B73 was chosen to establish an EMS mutant population. Its pollens were treated with a certain concentration of EMS that might harbor some base substitutions. Then these pollens were provided to the ear of B73, which yielded the M1. And then M1 were self-pollinated and obtained its ear named M2 (Figure 1A). The mutant phenotype is not visible in the M1 ears but will then reappear in the M2 ears, indicating that recessive mutants were produced. After establishing the B73 EMS mutant population, 670 mutants exhibited a diversity of kernel-defective phenotypes on the 12 DAP ear (Figure 1B). Among the kernel phenotypic mutants, 196 of them exhibit high ear set and neat kernel arrangement, with a segregation ratio (WT:MT) close to 3:1. We selected 20 of these mutants as experimental materials.

**Figure 2:**
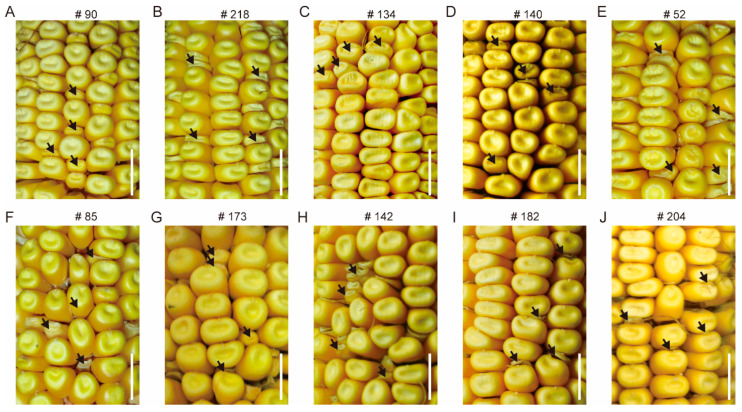
Classification of the phenotype of mature M3 kernels. (**A**,**B**): smk (small kernels); (**C**,**D**): sh (shrunk kernels); (**E**,**F**): mn (miniature seed); (**G**,**H**): dek (defective kernels); (**I**,**J**): emp (empty pericarp). The resulting kernels of self-pollinated M2. The black arrows represent the development of abnormal kernels. Scale bar = 10 mm.

**Figure 3:**
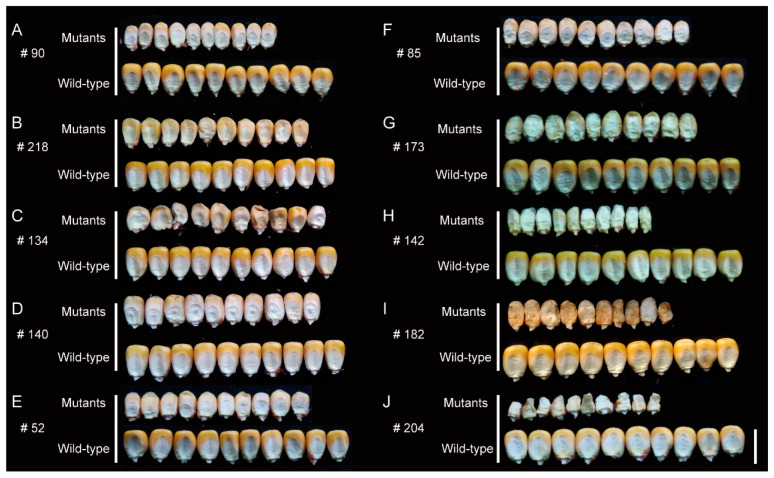
Details of the phenotype of mature M3 kernels. (**A**,**B**): smk (small kernels); (**C**,**D**): sh (shrunk kernels); (**E**,**F**): mn (miniature seed); (**G**,**H**): dek (defective kernels); (**I**,**J**): emp (empty pericarp). Comparisons of 10-kernel width between wild-type and its corresponding mutants. Scale bar = 10 mm.

These mutant phenotypes are not exactly the same; however, based on the characteristics of mature endosperm morphology of the M2 progeny, these mutants are classified as smk (small kernels), sh (shrunk kernels), mn (miniature seed), dek (defective kernels), and emp (empty pericarp) (Figure 2). After detailed observation, we found that mutants identified as smk exhibited relatively smaller kernels with regular shape, resembling the wild-type, and sh showed shrinkage at the top of the mutants. Similarly to the smk, mn kernels also displayed miniature kernels, but its pericarp is slightly loose. Dek kernels exhibited little endosperm, while emp kernels have almost no endosperm (Figure 3). However, endosperm development and filling, rather than embryo development, are important indicators to determine the classification of mutants. Therefore, well-developed and poorly developed embryos were observed concurrently in one type mutant; for example, #134 has endosperm similar to #140, but has obviously abnormal embryo.

To discover the causal genes that conferred the 20 mutants, we developed a novel approach named Easy Bulked sergeant RNA-seq Mapping (eBSRmap). We selected those M2 that have a segregation ratio of kernel to self-pollinated and chose WT kernels which contain heterozygote to develop a segregating M3. The pericarps of 12 DAP (days after pollination) M3 kernel were peeled off to eliminate influence of maternal genotypes. A total of 20 mutants and 20 relative wild-type siblings from the same self-pollinated M2 ear were sampled and formed two mixed pools, named mutant and wild-type pool, respectively (Figure 1C). Total RNA were extracted and constructed the RNA-seq libraries that were sequenced and produced at least 3 Gb of clean data. These high-quality data lay a robust foundation for subsequent analysis. The sequencing data were aligned to reference Genome (B73 RefGen_v4) to identify the SNP between the wild-type and mutants.

### 3.2. Discover Candidate Gene by SNP Calling

**Figure 4:**
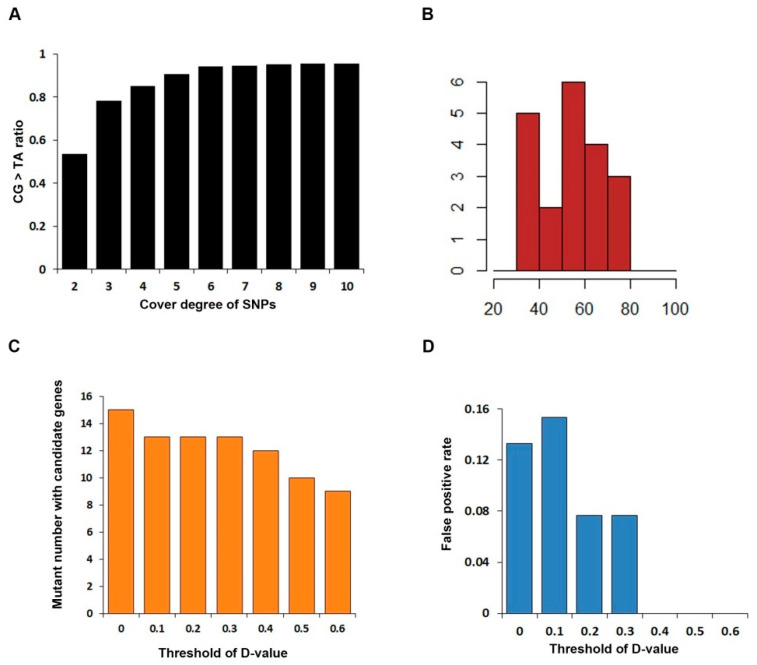
(**A**): Graph showing the proportion of CG > TA mutations as a function of SNP read coverage. (**B**): Frequency distribution of the number of mutants across different SNP counts. X-axis: number of SNPs; Y-axis: number of mutants. (**C**): Number of mutants containing candidate genes as a function of D-value thresholds. (**D**): False positive rate as a function of D-value thresholds.

To increase the correctness of SNPs, a rigorous pipeline to identify the EMS-induced mutations was adopted in this study. Since our samples are derived from the reference inbred line B73, theoretically, the vast majority of mutation sites occur independently, and the MAF (minor allele frequency) should be close to zero; SNPs with MAF greater than 10% were removed and SNPs detected in both mutants and its corresponding wild-type were retained for further study. Surprisingly, we summarized the correction between the ratio of base substitution from pyrimidine to purine and the SNP coverage, indicating that the SNP proportion of cytosine to thymine (C-to-T) and guanine to adenine (G-to-A) showed positive correlation until the coverage for one SNP is greater than five (Figure 4A). Therefore, the threshold of SNPs coverage is set as five to filter the SNPs believed to have high confidence. Furthermore, the number of SNPs for each mutant ranges from 30 to 80, with an average of 54 (Figure 4B).

To track the impact of these SNPs in 20 mutants, we were determined to annotate these SNPs to investigate each candidate variant for its effect on coding sequence by the analysis of SnpEff. As we expected, these SNPs were scattered in various regions of the genome, including promoters, exons, introns, and intergenic regions. However, the exons, especially coding sequences, contain the highest number of SNPs, accounting for 73.33% of the total SNPs. Among these exonic SNPs, synonymous mutations account for 30.56%, missense_variant account for 63.63%, stop-gained account for 3.66%, and splice_region_variant account for 1.89%.

### 3.3. Identification of SNP Markers Tightly Linked to the Mutant Gene

However, to detect which SNPs exhibit complete linkage disequilibrium with the causal gene, the SNP frequency, also named the SNP index (proportion of mutant reads in total reads under each SNP), for each mutant and its corresponding wild-type was calculated. In theory, the SNP index of the most linkage of SNP to phenotype is 100% and 33.33% for mutant and wild-type, respectively. Then, the linkage SNP were selected for further study.

Moreover, to further evaluate the degree of SNP linkage with the phenotype, D-value which represents the difference value of SNP index of mutant and wild-type was introduced to reflect the linkage relationship of SNPs and the mutant site. We paid our attention to the chromosome-covered linkage SNP location, calculated the D-value, and found that D-value of nearly 66.67% means high linkage. However, due to the limited number of individuals in the bulked pools, the actual D-values deviated from the theoretical value. To address this, we conducted simulated sampling statistics, calculating the frequency distribution of D-values on the causal gene in the bulked pools for 100,000 iterations. The results showed that the D-values corresponding to a confidence level of 0.95 was 0.5. As all our mutants are mono gene recessive, there must be false positive in mutants with more than one candidate gene. We calculated candidate gene number and false positive rate (the proportion of mutants with more than one candidate gene in all mutants) under different D-value thresholds (Figure 4C,D). Candidate gene number decreases as the threshold of D-value increases and the false positive rate becomes zero when D-value ≥ 0.4. Thus, based on the comprehensive analysis above—including the simulated sampling results reflecting the deviation in actual D-values from theoretical ones, as well as the trends of candidate gene number reduction and false positive rate elimination with increasing D-value thresholds—we decide to set the threshold to 0.4. On the other hand, the D-value is more accurate at SNPs with higher reads count. Under this law, we used an appropriate principle to identify the chromosome with the most linkage to the causative mutant site: SNPs with read count higher than 10 in both WT and MT pool, and D-value higher than 0.4. A total of 17 of 20 mutants identified the likely chromosome location. No tightly linked SNP was identified in five mutants (# 103, # 126, # 184, # 197, #229), and we listed the SNP most likely to be in linkage with the causal gene in each mutant (Table S1). However, none of these SNPs display an SNP index close to one, suggesting only weak linkage to the phenotype. In mutants # 103 and # 184, all above-threshold SNPs have coverage <10×, rendering their apparent linkage unreliable. For mutants #135, #205, and #206, above-threshold SNPs cluster on a single chromosome, whereas mutants #2, #32, and #119 show candidate peaks on several chromosomes. This dispersion indicates that our current filter still admits false positives; these spurious intervals were subsequently discarded by SNP-effect annotation (retaining only large-effect variants) (Figure 3).

Next, we screened SNPs on each likely chromosome location. We used the following pipeline to identify the SNP with the most linkage to the phenotype. (1) SNP index is greater than 0.9 in MT. (2) The D-value is higher than 0.4. (3) SNP was annotated as large effect (stop-gained and missense variation). Under this principle, 12 candidate genes were uncovered.

### 3.4. Confirmation of Candidate Genes

**Figure 5:**
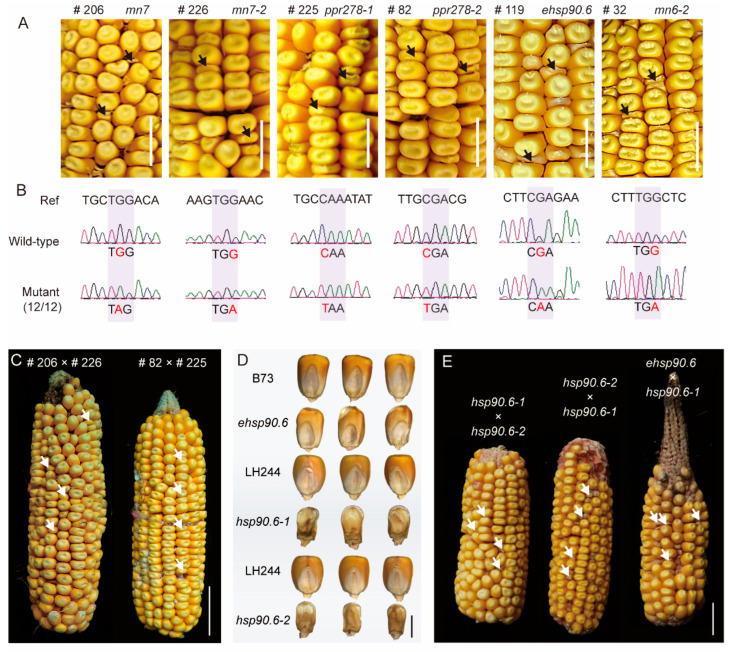
Validation of the candidate genes. (**A**) Phenotype of mutants and their corresponding candidate gene. The black arrows represent the development of abnormal kernels; (**B**) validation of co-separation by designing the primers for the location that is covered by the tightly linked SNP; (**C**) allelic test for *mn7* and *ppr278* which identified two alleles in our EMS libraries was performed. The white arrows represent the development of abnormal kernels; (**D**) phenotype of individual kernel derived from #119 and CRIPSR/Cas9 and their wild-type siblings; (**E**) validation of *ehsp90.6* by allelic test between different *ehsp90.6* mutants. The white arrows represent the development of abnormal kernels. Scale bar = 10 mm in (**A**), 20 mm in (**C**,**E**), and 5 mm in (**D**).

To test whether our candidate genes are reliable, we designed primers that spanned the mutant site located at the candidate gene. Then, 12 normal and 12 abnormal kernels from the M3 generation were selected; we peeled off their pericarp and extracted their DNA, respectively. If the abnormal kernels only can be detected by the mutant SNP, while the normal kernels only can be detected by the rare mutant SNP and major wild-type SNP, this kind of candidate genes will be recognized as genes that can be co-segregated with the phenotype. The results of sanger sequencing showed that eight out of twelve genes passed the analysis of co-segregation (Figure 5A,B. Figure S1 and Table S1). Among the four candidate genes that failed co-segregation validation, the SNP genotypes of #180 and #205 showed tight linkage with the phenotype: in the co-segregation test, 11 of the 12 individual plants of each mutant were homozygous for the mutant allele (MT). By contrast, mutants #2 and #254 displayed no linkage between genotype and phenotype.

Further confirmation of candidate genes will be carried out by focusing on genes that satisfy the condition for co-segregation.

Mutant genes of #105 and #43 had been reported by other teams before [26,27]. Mutants #206 and #226 in our library had small kernels and were mapped to the same causative gene *mn7*. It shows that they might be a pair of allelic mutants. Allelic test between #206 and #226 confirmed they were different mutant alleles of *mn7*. In addition, the allelic test validated that mutants #225 and #82 were controlled by the same conferred gene *PPR278* and further functional analysis revealed that *PPR278* functions in mitochondrial RNA splicing and editing (Figure 5C). Moreover, a mutant #32 was *mn6* (hereafter named *mn6-2*), an endoplasmic reticulum signal peptidase, which is allelic to the map-based cloned gene *mn6-1*. On the other hand, CRISPR/Cas9 technologies have been used in validating the phenotype and function of mutants. In our EMS library, the #119 mutant has the decreased kernel length, width, area, and weight compared with its wild-type, which is consistent with the CRISPR/Cas9 lines *hsp90.6-1* and *hsp90.6-2* (Figure 5D,E and Figure S2). The gene discovery work by eBSRmap, completed in 2018, discovered novel genes which was confirmed by map-based cloning in Lai lab after that [28,29,30,31].

## 4. Discussion

### 4.1. Advantages of eBSRmap

We found that eBSRmap applied to maize can rapidly identify finely mapped candidate genes for a given phenotype. In eBSRmap, we used EMS mutagenic pollen as M0 which pollinated to wild-type line used for mutagenesis followed by two subsequent selfing. As the mutant has been crossed back to its progenitor wild-type, the M2 progeny shows unequivocal segregation between the mutant and wild-type phenotypes. This contrasts with conventional crossing schemes for gene isolation that involve crosses between genetically distant lines. In distant crosses, the parent lines differ in many genes and obvious heterosis would strongly affect the phenotype. Our eBSRmap method does not introduce additional genetic backgrounds and conducts phenotypic identification in a relatively pure genetic background, thus achieving higher accuracy.

Whole-genome sequencing-based strategies like MutMap can be efficiently applied to crops with relatively small genomes. However, their application to crops with very large genomes—such as maize, sorghum, soybean, and barley—proves uneconomical. This is because MutMap requires relatively deep genome sequencing coverage (>10×), which becomes cost-prohibitive for large-genome species. Notably, our eBSRmap method addresses this limitation: it is specifically suitable for crops with large genomes. Unlike MutMap, eBSRmap does not rely on high-depth whole-genome sequencing, making it a more feasible and cost-effective choice for studying large-genome crops like the aforementioned maize, sorghum, soybean, and barley.

BSR-seq likewise leverages RNA-seq data for gene cloning. However, it requires a segregating population, and its mapping output is confounded by background genotype-by-environment interactions. In contrast, our approach employs EMS-generated near-isogenic mutants, which eliminates heterozygous segregation noise, markedly reduces false positives, and shortens the cloning timeline.

The data we used were derived from RNA-seq and can be simultaneously utilized for transcriptome analysis—such as differential expression analysis between wild-type and mutant samples—to help us understand the biological functions of mutant genes.

### 4.2. Potential Problems and Strategies for Improvement

In this study, more than half of the mutants (12 out of 20) failed to identify a credible mutant gene. Candidates for #180 and #205 showed tight linkage with the phenotype. For #2 and #254 the candidates were unlinked, yet potentially linked SNPs were detected on other chromosomes. In addition, #135, #152, and #256 also carried SNPs that may be tightly linked to the phenotype. Although the linked SNPs provided useful positional clues, eBSRmap in this study still failed to pinpoint the causal genes directly.

On the technical side, our internal benchmark shows that a minimum read depth of 5× is required to remove spurious SNPs while retaining true EMS-type changes. At the routinely used 1× cutoff, the canonical EMS spectrum (C->T, G->A) already exceeds 50%, indicating that many genuine mutations are being discarded. Raising depth to ≥5× would increase the number of reliable SNPs available for mapping and should markedly strengthen the linkage peaks.

On the biological side, as shown in Supplementary Figure S3, the mutant collection contains long chromosomal tracts (e.g., #32 on chr1) or even entire chromosomes (#43 on chr3) that are effectively marker-free because the EMS mutation density in our M2 material is far lower than in a typical F2 population. If the causal gene happens to lie in one of these deserts, the signal is inevitably weak or missing.

The other possible reasons for this may be the extremely low read coverage of certain gene transcripts in the transcriptome data due to the overabundance of a few gene transcripts. A total of 50 genes which had the highest expression form 68% of all data in the transcriptome. Thus, most data cannot provide efficient information for mapping. And a lot of low expression genes have not enough read coverage, this is probably one of reasons why we did not discover candidate genes in several mutants.

The aforementioned approaches including high-affinity oligonucleotide blocking and pseudo-random primer targeting can effectively increase the relative abundance of low-abundance transcripts in RNA-seq data [32,33]. By reducing the overrepresentation of highly abundant transcripts, these methods allocate more sequencing resources to low-abundance transcripts, directly addressing the issue of their under detection in conventional RNA-seq. This enhancement not only boosts the probability of detecting causal genes (which often exhibit low expression levels in many biological contexts) but also improves the SNP coverage of low-abundance transcripts, thereby increasing the overall marker density in the transcriptome and providing more robust molecular markers for downstream analyses such as functional validation.

In subsequent studies, we will integrate three key strategies to address current limitations: first, blocking highly expressed genes to reduce their interference in transcriptome data, thereby improving the detection sensitivity of low-abundance transcripts associated with causal genes; second, increasing sequencing depth to enhance the coverage of SNPs in candidate gene regions, especially for rare mutations that may be overlooked; third, applying the 10× MutMap method to detect cases where candidate genes are linked to mutant loci but do not co-segregate—this will help distinguish true causal genes from false positives caused by linkage drag. Through these approaches, we aim to identify the authentic causal genes and clarify the underlying reasons for the failure to detect causal genes in the present study. Additionally, we will further optimize the experimental workflow by evaluating the cost-effectiveness of each step (e.g., balancing the number of blocked high-expression genes with sequencing depth) and adjusting parameters such as D-value thresholds and pool construction strategies, ultimately establishing a more efficient, cost-effective, and higher-success-rate protocol for causal gene mining in large-scale mutant libraries.

## 5. Summary

This method can identify the causal genes of 40% of mutants in a mutant library, and this coverage makes it particularly valuable for conducting gene mining studies on large-scale mutant collections. Overall, this study establishes a rapid, cost-effective approach for gene mapping using a specialized material—EMS mutants—which are widely used in plant genetics, especially when working with large sets of mutant materials. By streamlining the mapping process, this method may facilitate the cloning of additional novel genes related to maize kernel traits; such progress could greatly advance our understanding of gene functions in maize, and potentially in other crops with similar scale of genomes.

## Data Availability

The original contributions presented in this study are included in the article/Supplementary Materials. Further inquiries can be directed to the corresponding authors.

## Supplementary Materials

The following supporting information can be downloaded at https://www.mdpi.com/article/10.3390/genes16111337/s1; Table S1. Most-linked SNP for each mutant; Figure S1. Validation of other two genes by the test of co-separation; Figure S2. Comparison the kernels from hsp90.6 mutants relative to wild-type; Figure S3. Manhattan plots of D-values for 20 mutants.
