# Supplementary material for: Mapping-by-Sequencing via eBSRmap (Easy Bulk Segregate RNA Mapping) in a B73 EMS Mutant Population
Source: Genes (Basel). 2025 Nov 6;16(11):1337. doi: 10.3390/genes16111337 (PMC12652423; doi:10.3390/genes16111337)
Supplement: Supplementary file 1 [file genes-16-01337-s001.zip › Table S1.pdf]

Table S1 Most-linked SNP for each mutant

Mutant ID	Chr	Position	SNP effect	D-value	SNP_Index of MT	Results of Mapping	Results of Validation
# 2	6	159774360	synonymous_variant	0.7879	0.9545	Non-Candidate Gene	
	5	74947478	missense_variant	0.4000	1.0000	Candidate Gene	Non-Linkage
# 32	6	142178709	stop_gained	0.8492	1.0000	Candidate Gene	Cosegregate
# 43	5	3927736	missense_variant	0.5960	0.9842	Candidate Gene	Cosegregate
# 82	5	77317095	stop_gained	0.6765	1.0000	Candidate Gene	Cosegregate
	5	68367208	synonymous_variant	0.6458	1.0000	Non-Candidate Gene	
# 105	1	276804400	stop_gained	0.9000	0.9000	Candidate Gene	Cosegregate
# 119	3	134956954	missense_variant	0.5769	0.9963	Candidate Gene	Cosegregate
# 135	2	196143595	synonymous_variant	0.7647	1.0000	Non-Candidate Gene	
# 152	4	25998691	synonymous_variant	0.8000	1.0000	Non-Candidate Gene	
# 180	8	14484080	stop_gained	0.7167	0.9412	Candidate Gene	Tight linkage
	1	276303266	missense_variant	0.7167	0.9167	Non-Candidate Gene	
	8	26156137	missense_variant	0.4635	0.9250	Non-Candidate Gene	
# 205	3	96165217	missense_variant	1.0000	1.0000	Non-Candidate Gene	
	5	41112697	missense_variant	0.4074	1.0000	Candidate Gene	Tight linkage
# 206	2	31126061	synonymous_variant	0.8438	0.9390	Non-Candidate Gene	
	2	50703343	stop_gained	0.6667	1.0000	Candidate Gene	Cosegregate
# 225	5	67132854	synonymous_variant	0.7778	1.0000	Non-Candidate Gene	
	5	77317230	stop_gained	0.6250	1.0000	Candidate Gene	Cosegregate
# 226	2	50703170	stop_gained	0.8371	0.9583	Candidate Gene	Cosegregate
# 254	9	107407495	synonymous_variant	1.0000	1.0000	Non-Candidate Gene	
	7	2079029	stop_gained	0.8010	0.9492	Candidate Gene	Non-Linkage
# 256	2	149554785	synonymous_variant	1.0000	1.0000	Non-Candidate Gene	
	5	9534717	synonymous_variant	1.0000	1.0000	Non-Candidate Gene	
# 126	2	174592119	missense_variant	0.5304	0.8161	weak linkage	
# 103	1	252795023	missense_variant	0.3627	0.8333	weak linkage	
# 229	9	51048979	splice_region_variant	0.4202	0.7143	weak linkage	
# 197	3	21322181	missense_variant	0.3203	0.5556	weak linkage	
# 184	8	6153309	missense_variant	-0.0450	0.5865	seem Non-Linkage	
