# Supplementary figures and images for: Mapping-by-Sequencing via eBSRmap (Easy Bulk Segregate RNA Mapping) in a B73 EMS Mutant Population
Source: Genes (Basel). 2025 Nov 6;16(11):1337. doi: 10.3390/genes16111337 (PMC12652423; doi:10.3390/genes16111337)

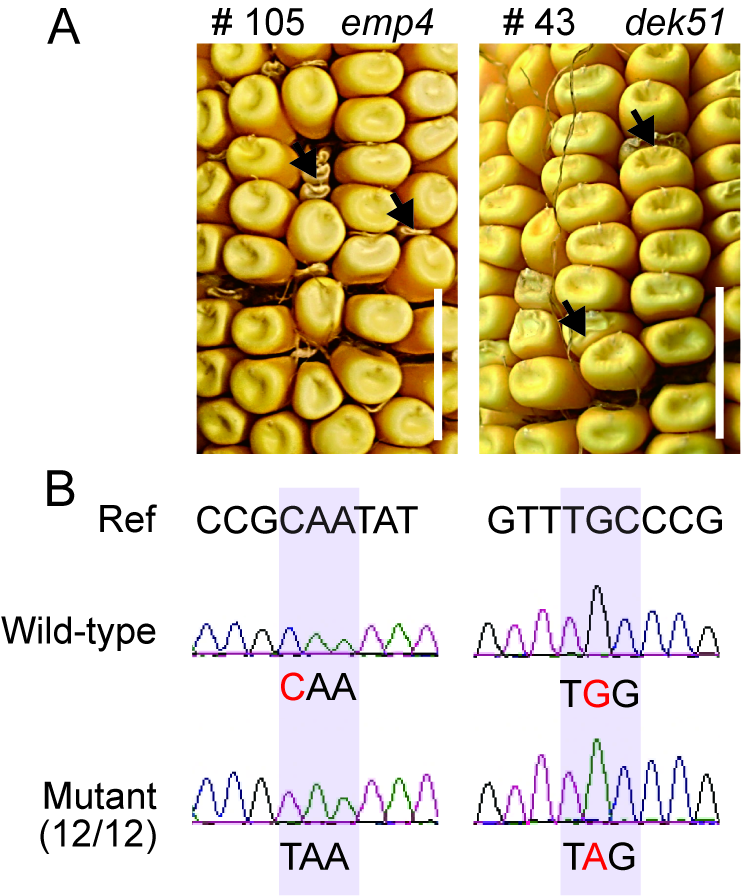

Supplement: Supplementary file 1 [file genes-16-01337-s001.zip › FigureS1.tif]

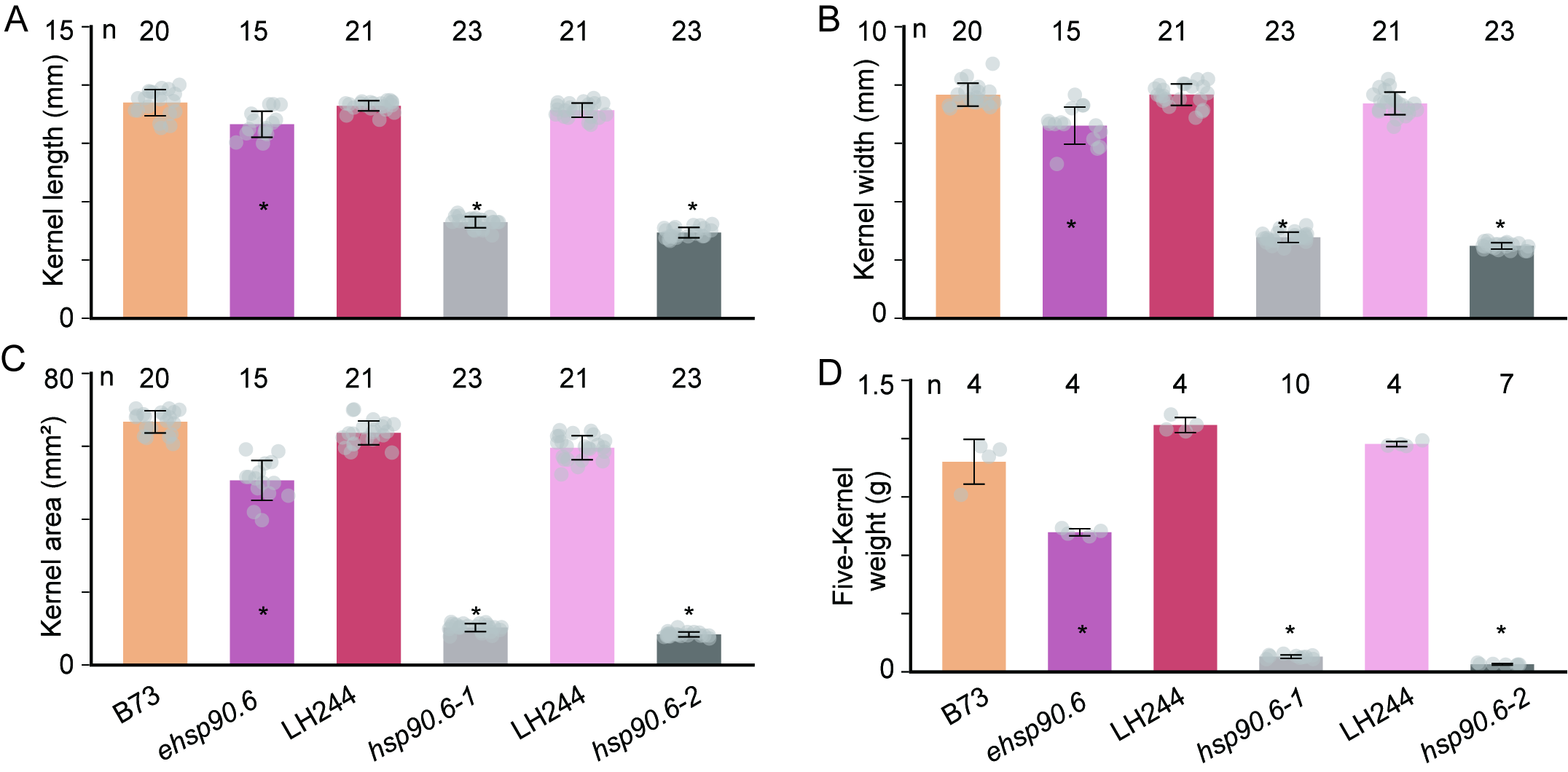

Supplement: Supplementary file 1 [file genes-16-01337-s001.zip › FigureS2.tif]

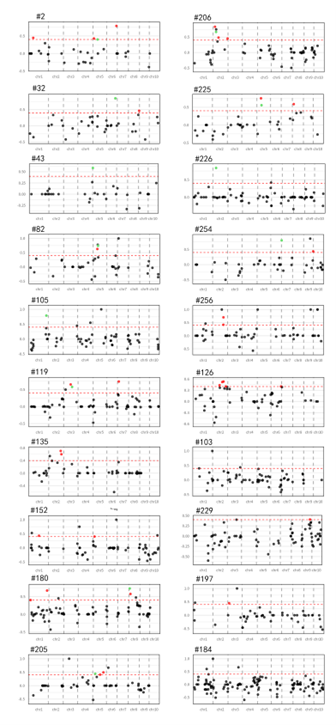

Supplement: Supplementary file 1 [file genes-16-01337-s001.zip › FigureS3.tif]
